# miR-564 inhibits hepatocellular carcinoma cell proliferation and invasion by targeting the GRB2-ERK1/2-AKT axis

**DOI:** 10.18632/oncotarget.22504

**Published:** 2017-11-18

**Authors:** Chaojie Liang, Yingchen Xu, Hua Ge, Bingchen Xing, Guanqun Li, Guangming Li, Jixiang Wu

**Affiliations:** ^1^ Department of General Surgery, Beijing Tongren Hospital, Capital Medical University, Dongcheng, Beijing 100730, China

**Keywords:** miR-564, HCC, GRB2, PI3K/AKT, ERK1/2

## Abstract

Recent studies have shown that miR-564 is closely related to the development of various tumors, including breast cancer, lung cancer and glioma. However, few studies have examined miR-564 in hepatocellular carcinoma (HCC). Here, we demonstrated that miR-564 expression in HCC tissues was lower than that in adjacent noncancerous tissues and that miR-564 expression was associated with tumor size, tumor number and vein invasion. Bioinformatics analyses showed that low levels of miR-564 were correlated with poor prognosis. Furthermore, upregulation of miR-564 impaired SMCC7721 and MHCC97H cell proliferation, migration and invasion *in vitro* and reduced tumorigenesis *in vivo*. Next, we found that GRB2 was a direct target gene of miR-564 in the HCC cell lines. GRB2 was highly expressed in HCC tissues and negatively correlated with miR-564 expression levels. When GRB2 was downregulated by GRB2-siRNA, HCC cell proliferation, invasion and metastasis were impaired, and restoring GRB2 expression partially reversed the inhibitory effects of miR-564. Western blot analysis showed that miR-564 overexpression reduced GRB2 expression in HCC cell lines and inhibited ERK1/2 and AKT phosphorylation. miR-564 overexpression also upregulated the epithelial-like cell marker E-cadherin and downregulated the interstitial cell-like markers N-cadherin and vimentin. These results suggest that miR-564 inhibits the malignant phenotype of HCC cells by targeting the GRB2-ERK1/2-AKT axis. Consequently, miR-564 may be used as a prognostic marker and therapeutic target for HCC.

## INTRODUCTION

Hepatocellular carcinoma (HCC) is one of the most common malignancies in the world. GLOBALCAN data show that the number of new cases of global HCC was 782,000 in 2012; furthermore, HCC has an incidence rate of 83% in non-developed areas and 50% in China [1]. China is an area of high incidence of liver cancer. In fact, the incidence of liver cancer in 2015 was 466,100, and the number of deaths was 422,000 [2]. In recent years, the treatment of liver cancer has greatly improved due to continuous improvements in surgical treatments, modern imaging technology and individualized treatment plans. However, the prognosis of patients with liver cancer is still not satisfactory. The main reasons for the poor prognosis of HCC is that early diagnosis of liver cancer is difficult and that liver cancer is prone to recurrence and metastasis [3–5]. Furthermore, there is a lack of biomarkers for early diagnosis and prognosis prediction. Therefore, the molecular mechanisms underlying HCC metastasis and biomarkers for early diagnosis are urgently needed to develop more effective therapies for HCC.

MicroRNAs (miRNAs) are single-stranded, non-coding small RNAs that are approximately 20–24 nucleotides in length and play a regulatory role in the expression of many protein-coding genes [6, 7]. miRNAs often target the non-coding 3′-UTR of mRNAs. If an miRNA is not fully matched to the mRNA, translation will be inhibited; however, when there is a complete match, the mRNA will be cut or cleaved [8]. This process leads to decreased protein levels and may cause serious diseases. Although miRNAs account for 1–3% of the human genome, they regulate the expression of more than 30% of the genes [9]. There is growing evidence that abnormal miRNA expression is closely related to tumorigenesis as well as tumor development, recurrence and metastasis; additionally, miRNAs may be a new marker for predicting tumorigenesis and effective tumor treatments [10]. Various miRNAs are involved in the occurrence and development of HCC; for example, miR-21 is overexpressed in liver cancer, and downregulation of miR-21 inhibits tumor cell proliferation, migration and invasion [11]. However, miR-150 [12] is downregulated in HCC, and overexpression of miR-150 inhibits tumor cell proliferation and invasion. As GAB1 is a target gene of miR-150, miR-150 overexpression inhibits the function of GAB1 to suppress epithelial-mesenchymal transition (EMT) in tumor cells.

miR-564 expression is downregulated in gastric cancer [13], lung cancer [14], breast cancer [15] and glioblastoma [16], and miR-564 overexpression impairs tumor cell proliferation, migration and invasion. However, miR-564 targets different genes in different tumors, indicating that miR-564 may play different roles in different tumors. Currently, no study has investigated the relationship between miR-564 and HCC. The aim of this study was to investigate the role of miR-564 in the development of HCC. Our results showed that miR-564 expression was downregulated in HCC compared to adjacent noncancerous tissues. Moreover, miR-564 overexpression inhibited HCC proliferation, invasion and metastasis by targeting the GRB2-ERK1/2-AKT axis. These data may provide a theoretical basis for using miR-564 in the diagnosis and treatment of HCC.

## RESULTS

### miR-564 expression is downregulated in HCC tissues and is associated with the clinicopathological characteristics and prognosis of patients with HCC

**Figure 1:**
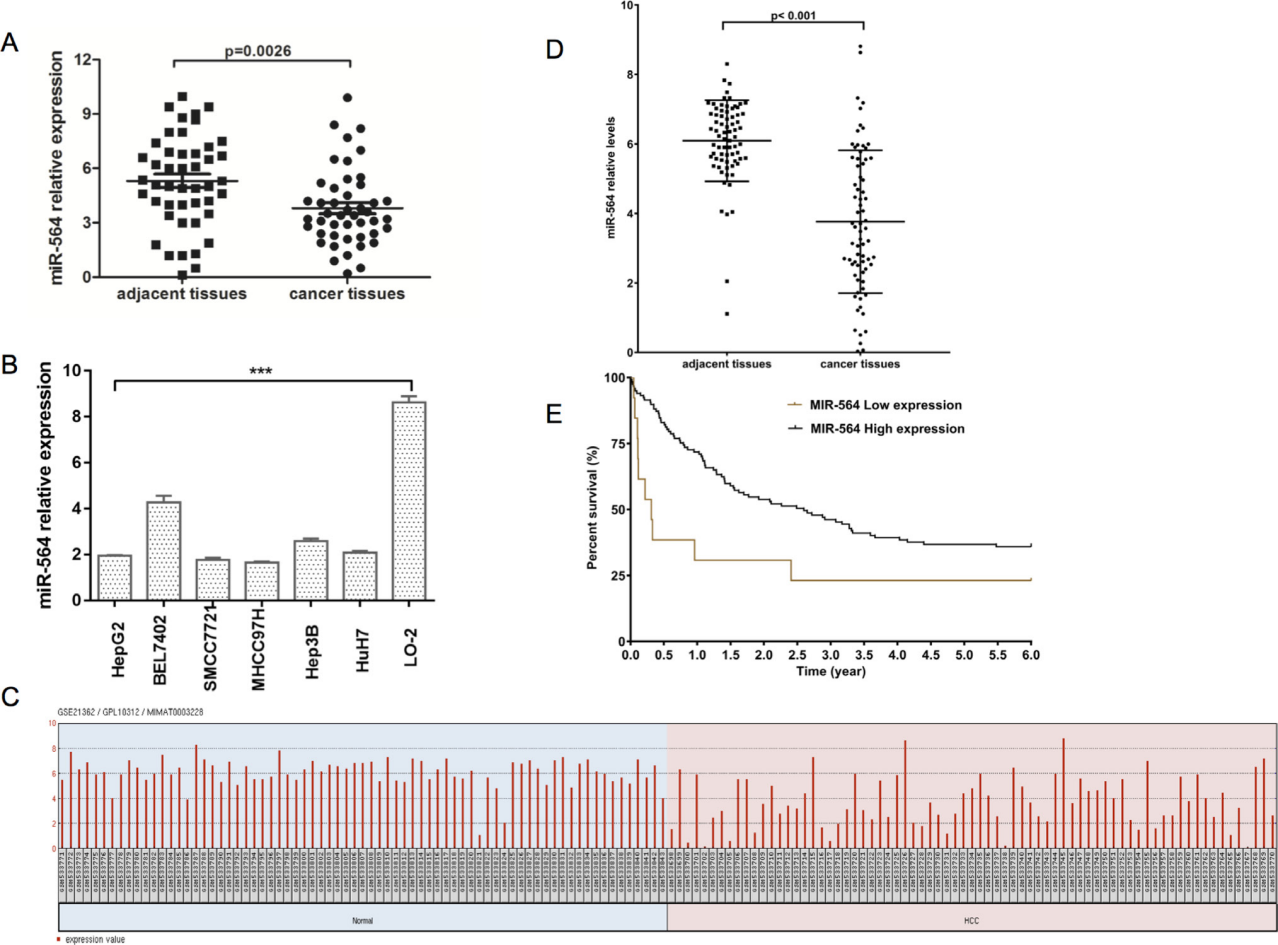
miR-564 expression is downregulated in HCC (**A**) miR-564 expression in HCC tissues and adjacent noncancerous tissues was quantified by qRT-PCR. The differences were statistically significant. (**B**) The expression of miR-564 in different HCC cell lines and the normal liver cell line LO-2 was measured by qRT-PCR; U6 was used as an internal reference. (**C**) miR-564 data were collected from the GEO dataset GSE21362. After quality control, 73 HCC and adjacent noncancerous liver samples were used for the analysis. (**D**) Differentially expressed levels of miR-564 in the GSE14520 dataset were determined using the Limma package on the R platform. The cutoff values for significantly differentially expressed miR-564 were a *P* value < 0.01 (Student's *t*-test) and |lgFC| > 1 (fold change). (**E**) HCC patient survival was analyzed using data downloaded from TCGA database. A total of 130 HCC patients were included in the survival analysis. These patients were divided into two groups, the miR-564-low and miR-564-high groups, based on their miR-564 expression levels. miRNA and gene expression levels were measured using the Limma package on the R platform. The cutoff threshold was set to separate the top 10% of the miR-564-low patients from the top 90% of the miR-564-high patients. Survival curves were estimated using the Kaplan–Meier method and compared using the log-rank test. Data in the bar graphs represent the mean ± SD of three repeated experiments.

First, we compared the expression of miR-564 in HCC tissues and adjacent noncancerous tissues from 46 patients by qRT-PCR. U6 was used as the internal standard. As shown in Figure 1A, miR-564 expression was significantly downregulated in HCC tissues compared with adjacent tissues (*P <* 0.05). To further evaluate the relationship between miR-564 expression levels and clinicopathological parameters of patients with HCC, 46 patients were divided into two groups according to the median miR-564 expression level. As shown in Table 3, we collected the clinicopathological characteristics of all the patients. miR-564 expression levels were correlated with tumor size (≥5 cm), tumor number and vein invasion. However, there was no relationship between the expression level of miR-564 and sex, age, cirrhosis, tumor differentiation or TNM stage. As shown in Figure 1C–1D, we also analyzed miR-564 expression in 73 samples of HCC and adjacent noncancerous tissues in the GSE21362 dataset by bioinformatics. We demonstrated that miR-564 is downregulated in tumor tissues compared with normal liver tissues, which is consistent with the results obtained using our database (*P <* 0.05). As shown in Figure 1E, Kaplan–Meier survival analysis showed that low levels of miR-564 expression indicated a poor prognosis (*P <* 0.05). These results suggest that miR-564 may be involved in the malignant progression of HCC.

### miR-564 inhibits SMCC7721 and MHCC97H cell proliferation, migration and invasion *in vitro* and *in vivo*

**Figure 2:**
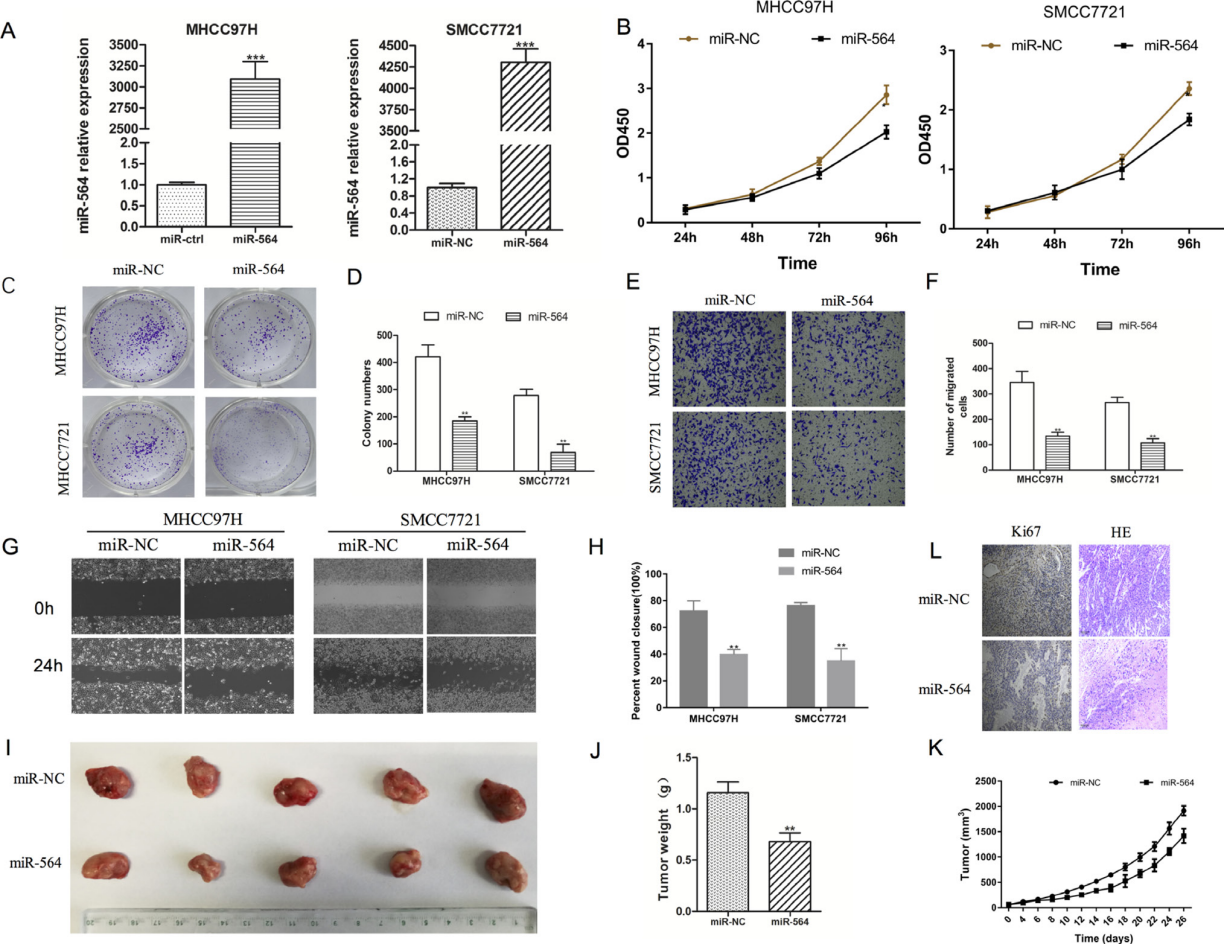
miR-564 inhibits SMCC7721 and MHCC97H cell proliferation, migration and invasion *in vitro* (**A**) miR-564 expression in the miR-564 group and miR-NC group as measured by qRT-PCR. U6 was used as a loading control. There was a significant increase in the expression level of miR-564 in the miR-564 group (*P <* 0.001). (**B**) Cell viability in various groups as measured by the Cell Counting Kit-8 (CCK-8) assay. Cell viability was markedly suppressed in the miR-564 groups in a time-dependent manner (*P <* 0.05). (**C**–**D)**. Clonogenic ability of the cells in various groups as measured by the colony formation assay. The clonogenic ability of the miR-564 groups was significantly decreased compared with that of the miR-NC groups (*P <* 0.05). (**E**–**F**) Cell migration as indicated by the Transwell migration assay. The number of cells that migrated into the lower chamber was significantly lower in the miR-564 groups than in the miR-NC groups (*P <* 0.05). (**G**–**H**) Wound healing assays indicated that cell migration was impaired in the miR-564 group (*P <* 0.05). (**I**) Tumor volume was measured after cells were subcutaneously injected into the right posterior flank area of nude mice. The tumor volumes were smaller in the miR-564 groups than in the NC group. (**J**) Tumor weights in the miR-564 groups were lower than those in the NC groups. (**K**) Growth curves indicated that the rate of tumor formation in the miR-564 groups was slower than that in the NC groups. (**L**) Ki-67 immunohistochemical staining and H&E staining of tumors from the miR-564 groups and NC groups. All assays were repeated three times, and the mean values were used for comparisons.

Next, we explored the molecular mechanism of miR-564 in HCC cell lines. First, we examined the expression of miR-564 in six HCC cell lines (HepG2, SMCC7721, MHCC97H, BEL-7402, Hep3B and Huh-7) and a normal liver cell line (LO-2) by qRT-PCR. As shown in Figure 1B, the expression level of miR-564 in the HCC cell lines was significantly lower than that in the LO-2 cell line (*P <* 0.05). miR-564 expression was the lowest in the SMCC-7721 and MHCC97H cell lines; therefore, they were used in subsequent experiments. A miR-564-overexpressing lentiviral vector was successfully transfected into the HCC cell lines (Figure 2A). SMCC-7721 and MHCC97H cells were each divided into two groups: the SMCC-7721-miR-NC and SMCC-7721-miR-564 groups; and the MHCC97H-miR-NC and MHCC97H-miR-564 groups, respectively. miR-564 expression was significantly increased in the miR-564 groups compared to that in the NC groups according to qRT-PCR (*P <* 0.05).

To determine the effect of miR-564 on HCC cell proliferation, we used the CCK-8 and colony formation assays. As shown in Figure 2B, proliferation was significantly inhibited in the miR-564 group after the second day (48 h) and was lower than that in the NC group (*P <* 0.05). As shown in Figure 2C–2D, the colony formation assays indicated that cells in the miR-564 groups formed fewer colonies compared with those in the NC groups (*P <* 0.05). These results suggest that miR-564 overexpression inhibits HCC cell proliferation. Then, we examined the effect of miR-564 on HCC cell migration and metastasis using Transwell and wound healing assays. As shown in Figure 2E–2F, the results of the Transwell assays showed that the number of cells that traveled through the polycarbonate membrane was significantly reduced in the miR-564 groups compared with that in the NC groups (*P <* 0.05). Similarly, in the wound healing assay (Figure 2G–2H), miR-564 overexpression inhibited HCC cell proliferation, invasion and metastasis *in vitro*.

To observe the effects of miR-564 on HCC proliferation *in vivo*, MHCC97H-miR-564 or MHCC97H-miR-NC cells were subcutaneously injected into nude mice. As shown in Figure 2I–2J, tumor formation (tumor weight) in the miR-564 groups was significantly suppressed compared to that in the NC groups (*P <* 0.05). Throughout the experiment, the tumor growth rate in the miR-564 groups was significantly slower than that in the NC groups (*P <* 0.05) (Figure 2K). After removing the tumor tissue, hematoxylin and eosin (H&E) staining was performed to confirm the formation of subcutaneous tumors in nude mice; additionally, Ki-67 immunohistochemical staining was significantly weaker in the miR-564 groups than in the control groups (Figure 2L). These results confirm that miR-564 overexpression significantly inhibited HCC proliferation *in vivo*.

### GRB2 is a direct target of miR-564 in hepatocellular cells, and silencing GRB2 inhibits HCC cell proliferation and invasion

**Figure 3:**
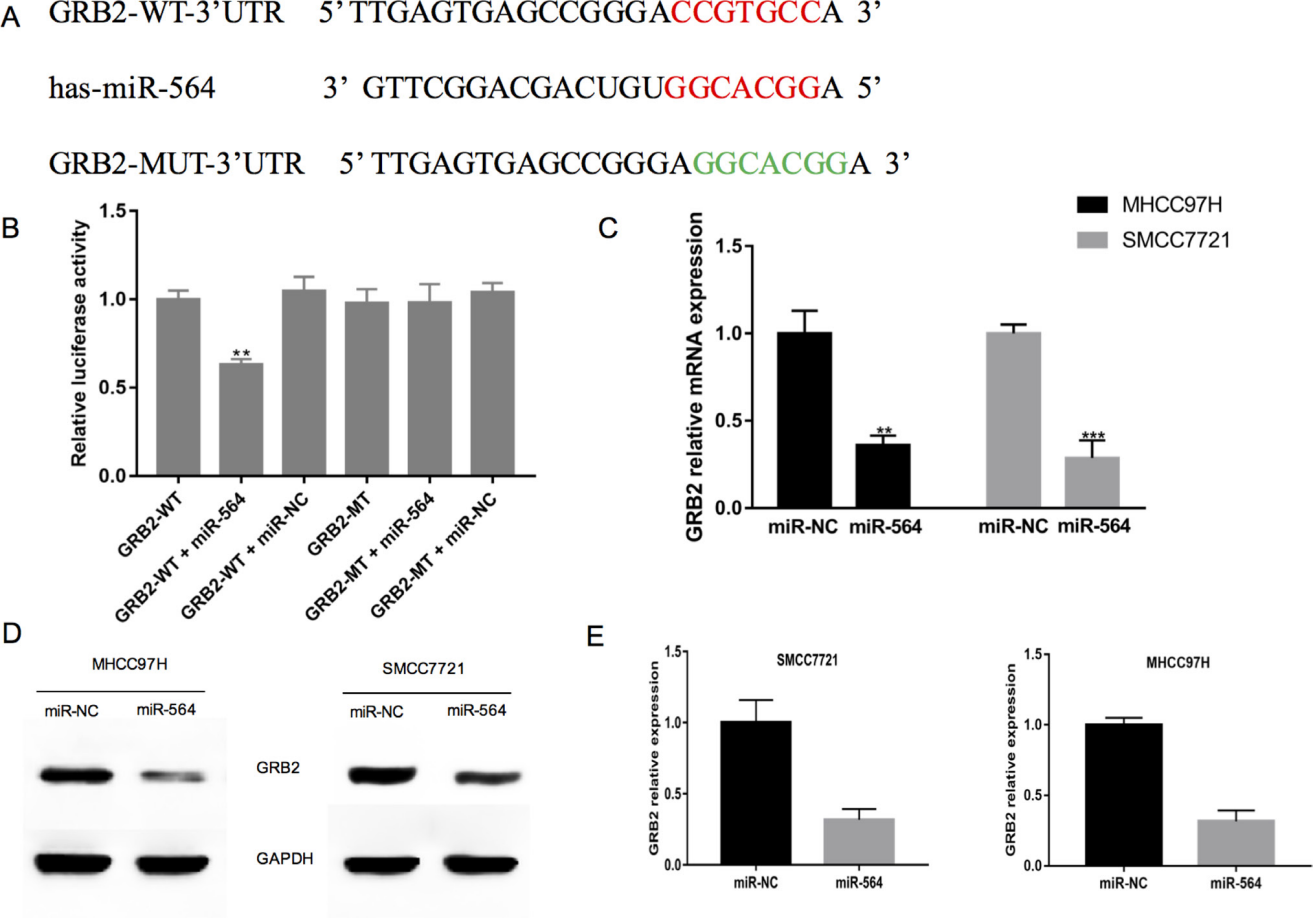
GRB2 is a direct target of miR-564 in hepatocellular cells (**A**) The predicted miR-564 binding site in the 3′-UTR of GRB2 and the mutated 3′-UTR of GRB2, which was generated using the complementary sequence in the seed region of miR-564. (**B**) miR-564 or NC and a luciferase vector encoding the WT or MUT GRB2 3′-UTR were transfected into 293T cells, and the relative luciferase activity was measured. (**C**) GRB2 mRNA expression in the MHCC97H and SMCC7721 cells of the miR-564 and miR-NC groups as detected by qRT-PCR. (**D**–**E**) GRB2 protein expression in the MHCC97H and SMCC7721 cells of the miR-564 and miR-NC groups as detected by western blotting. All assays were repeated three times, and the mean values were used for comparisons.

Next, we used the bioinformatics software TargetScan, miRTarBase and DIANA to predict the possible target genes of miR-564. After analyzing the predicted results, we chose GRB2 for subsequent experiments. As shown in Figure 3A, bioinformatics analyses showed that the GRB2 3′-UTR contained a miR-564 binding site. To verify whether GRB2 is a direct target gene of miR-564, we constructed luciferase reporter gene vectors with the wild-type and mutant 3′-UTR of GBR2 (GRB2-WT-3′-UTR and GRB2-MUT-3′-UTR). Then, we co-transfected 293T cells with miR-564 mimic or NC and GRB2-WT-3′-UTR or GRB2-MUT-3′-UTR. As shown in Figure 3B, luciferase activity was significantly decreased 48 h after co-transfection with the GRB2-WT-3′-UTR reporter vector and miR-564 mimic (*P <* 0.05). However, there were no obvious changes in luciferase activity when the GRB2-MUT-3′-UTR and miR-564 mimic were co-transfected. These results suggest that miR-564 binds to the GRB2 3′-UTR and that GRB2 may be a direct target gene of miR-564. To further verify that GRB2 is a target gene of miR-564, we used qRT-PCR to measure the GRB2 mRNA levels in MHCC97H and SMCC7721 cells when miR-564 was overexpressed (Figure 3C). miR-564 overexpression significantly reduced GRB2 mRNA levels (*P <* 0.05), and similar results were shown by western blot (Figure 3D–3E). The above results indicate that miR-564 negatively regulates GRB2 expression and that GRB2 is a direct target gene of miR-564.

**Figure 4:**
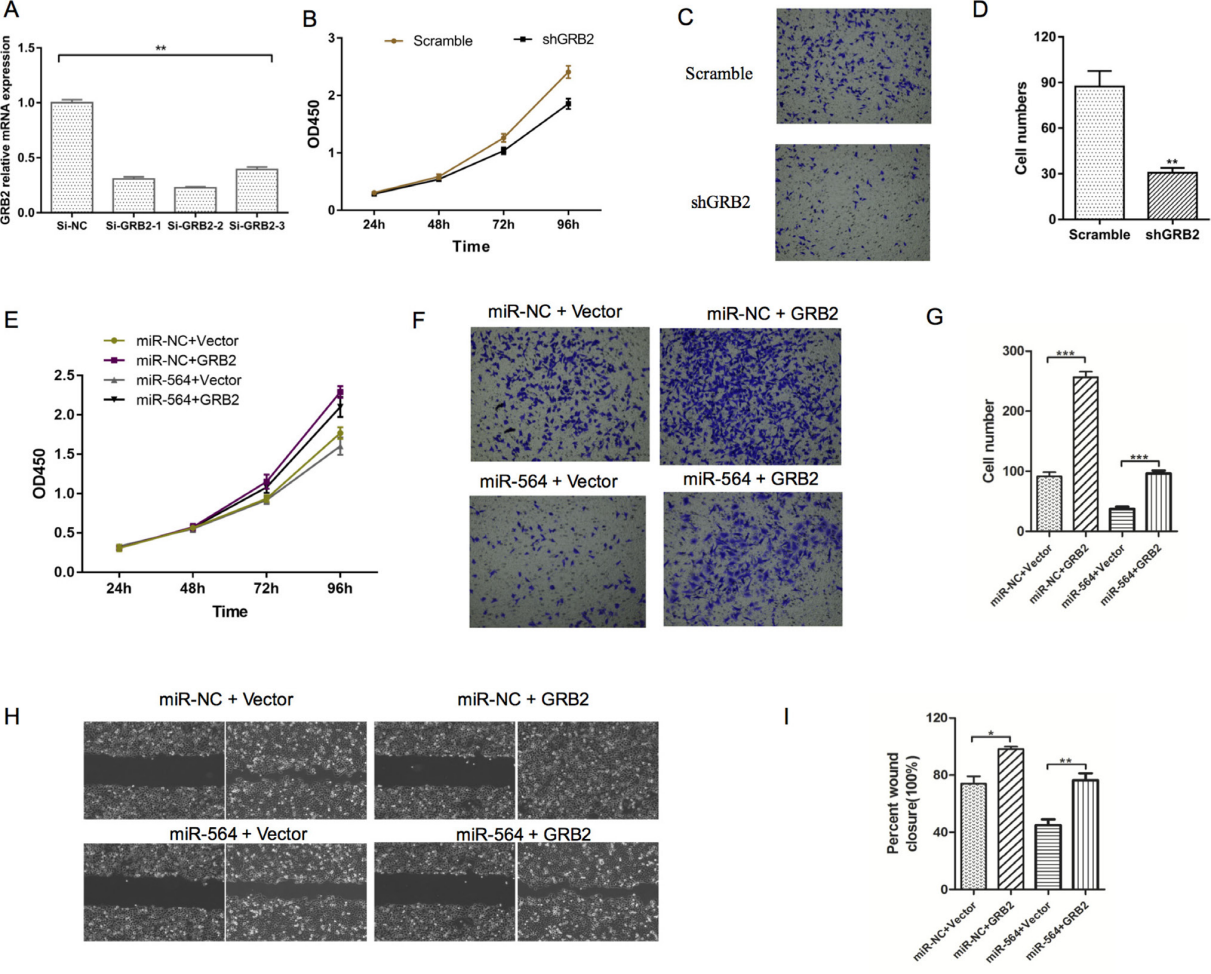
Silencing GRB2 inhibits HCC cell proliferation and invasion (**A**) GRB2 mRNA expression was significantly decreased in the MHCC97H cells of the si-GRB2 groups compared to those of the si-NC groups. si-GRB2 was chosen for subsequent experiments. (**B**) Cell viability in the sh-GRB2 and sh-NC (scramble) groups was measured by the CCK-8 assay. (**C**–**D**) Cell migration in the sh-GRB2 and scramble groups was measured by the Transwell assay. (**E**) Cell viability in the different groups was measured by the CCK-8 assay. (**F**–**G**) Cell migration in the different groups was measured by the Transwell assay. (**H**–**I**) Results of the wound healing assay. All assays were repeated three times, and the mean values were used for comparisons. ^*^P < 0.05, ^**^P < 0.01 and ^***^P < 0.001.

To determine whether miR-564 inhibited HCC proliferation, invasion and metastasis by regulating GRB2, we transfected GRB2-siRNA and GRB2-NC into MHCC97H cells. As shown in Figure 4A, GRB2 mRNA levels in the GRB2-siRNA group were significantly lower than those in the GRB2-NC group (*P <* 0.05). Moreover, cell proliferation, invasion and migration were impaired in the GRB2-siRNA group compared to the GRB2-NC group (*P <* 0.05) as determined by the CCK-8 and Transwell assays (Figure 4B–4D). These results demonstrated that downregulating GRB2 mimicked the inhibitory effects of miR-564 on hepatoma cell functions. Then, we constructed a GRB2 overexpression vector without the 3′-UTR. The GRB2 overexpression plasmid and the blank control plasmid were transfected into the miR-564 and miR-NC groups, respectively. As shown in Figure 4E, miR-564 inhibited hepatoma cell proliferation, and restoration of GRB2 expression reversed the inhibitory effects of miR-564 (*P <* 0.05). In addition, as shown in Figure 4F–4I, Transwell and wound healing assays showed that miR-564 overexpression inhibited invasion and migration in hepatocarcinoma cells. After restoring GRB2 expression in MHCC97H miR-564 cells, cell invasion and migration were increased, and the inhibitory effects of miR-564 were partially blunted. These results further confirmed that miR-564 inhibited hepatocarcinoma cells from forming malignant tumors by targeting GRB2.

### GRB2 is upregulated in HCC and is inversely correlated with miR-564 expression

**Figure 5:**
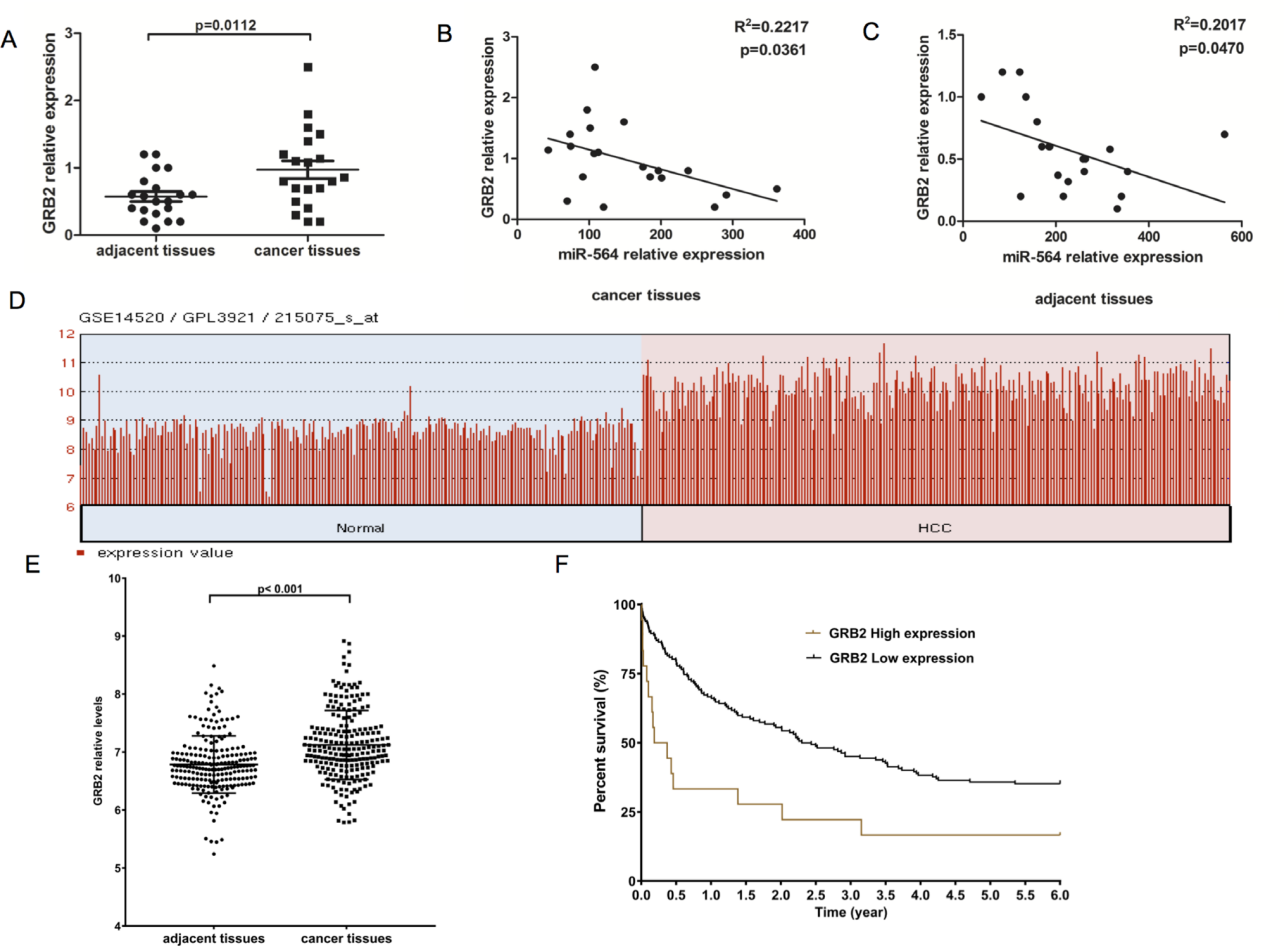
GRB2 is upregulated in HCC and is inversely correlated with miR-564 expression (**A**) GRB2 mRNA expression in 20 HCC and adjacent noncancerous tissues as detected by qRT-PCR. (**B**–**C**) The correlation between miR-564 and GRB2 mRNA expression levels in cancer tissues and adjacent tissues was determined by Spearman's correlation analysis. (**D**–**F)**. GRB2 was differentially expressed in the GSE14520 dataset, as determined using the Limma package on the R platform. The cutoff for significantly differentially expressed levels of miR-564 was *P* value < 0.01 (Student's *t*-test) and |lgFC| > 1 (fold change). HCC patient survival was analyzed using data downloaded from TCGA database. A total of 180 HCC patients were included in the survival analysis. These patients were divided into two groups, the GRB2-low and GRB2-high groups, based on their GRB2 expression levels. Gene expression levels were measured using the Limma package on the R platform. The cutoff threshold was set to separate the top 10% of the GRB2-high patients from the top 90% of the GRB2-low patients. The survival curves were estimated using the Kaplan–Meier method and were compared using the log-rank test.

To further verify that miR-564 directly regulates GRB2, we measured GRB2 expression levels in 20 pairs of HCC tissues and adjacent noncancerous tissues by qRT-PCR. As shown in Figure 5A, the GRB2 mRNA levels in HCC were significantly increased. We found the same results using the GSE14520 dataset, and high levels of GRB2 predicted a poor prognosis in the TCGA dataset (*P <* 0.05) (Figure 5D–5F). In addition, as shown in Figure 5B–5C, miR-564 expression in HCC was negatively correlated with GRB2 expression according to Spearman correlation analyses (*R*^2^ = 0.2217, *P* = 0.0361 in cancer tissues; *R*^2^ = 0.2017, *P* = 0.047 in adjacent tissues).

### miR-564 regulates signaling pathways downstream of GRB2

**Figure 6:**
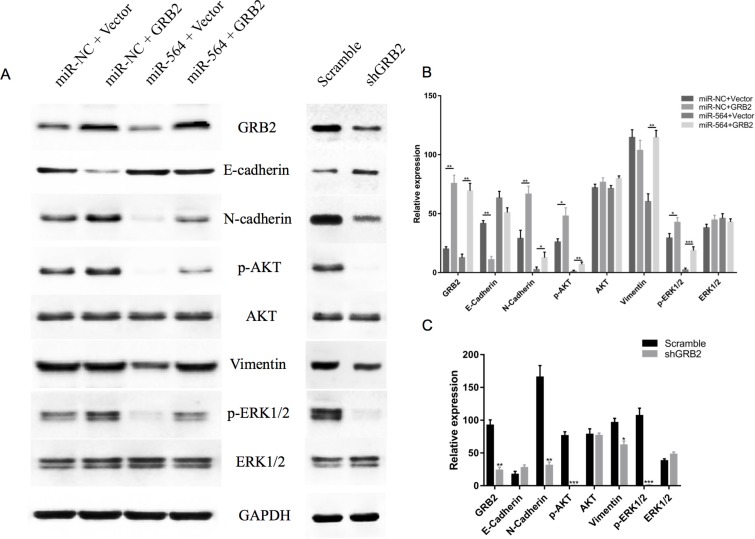
miR-564 regulates signaling pathways downstream of GRB2 (**A**) Expression levels of GRB2, E-cadherin, N-cadherin, p-AKT, AKT, vimentin, p-ERK1/2 and ERK1/2 in the miR-NC + vector, miR-NC + GRB2, miR-564 + vector, miR-564 + GRB2, GRB2-scramble and shGRB2 groups were measured by western blot. (**B**–**C**) The ratio of each protein to GAPDH. All assays were repeated three times, and the mean values were used for comparisons. ^*^P < 0.05, ^**^P < 0.01 and ^***^P < 0.001.

To further study the regulatory mechanisms of miR-564 in HCC, we analyzed the phosphorylation levels of PI3K/AKT and MAPK, which are downstream signaling molecules of GRB2. As shown in Figure 6A–6C, when miR-564 was overexpressed in MHCC97H cells, the expression levels of GRB2 and the phosphorylation levels of ERK1/2 and AKT were significantly decreased (*P <* 0.05). However, the total ERK1/2 and AKT levels (*P <* 0.05) and the phosphorylation levels of ERK1/2 and AKT were upregulated when GRB2 was overexpressed. These data indicate that restoring GRB2 expression partially reverses the effects of miR-564 on ERK1/2 and AKT.

Next, we examined changes in EMT markers after miR-564 overexpression in cells. As shown in Figure 6A–6C, the expression level of epithelial-like cell marker E-cadherin was upregulated, and the expression levels of interstitial-like cell molecule markers N-cadherin and vimentin were downregulated. These results suggest that miR-564 overexpression inhibits the EMT process in HCC and that restoring GRB2 expression reverses the inhibitory effects of miR-564. After silencing GRB2, we observed results similar to those when miR-564 is overexpressed.

The above results indicate that miR-564 inhibits HCC cell proliferation and invasion by targeting GRB2 and downregulating AKT and ERK1/2 signaling.

## DISCUSSION

In this study, we explored the relationship between miR-564 levels and HCC. Our results demonstrate that miR-564 was downregulated in HCC and that low expression levels of miR-564 were closely related to tumor size, tumor number and vein invasion, which are indicators of poor prognosis. Additionally, we showed that miR-564 was downregulated in HCC cell lines and that SMCC7721 and MHCC97H cell proliferation, invasion and metastasis were impaired by miR-564 overexpression *in vivo* and *in vitro*. Then, we demonstrated that GRB2 was a direct target gene of miR-564 in HCC by bioinformatics analyses and luciferase assays. GRB2 was highly expressed in HCC tissue and negatively correlated with the expression of miR-564; additionally, high GRB2 expression levels were associated with poor prognosis based on analyses of TCGA database. The effects of GRB2 knockdown were similar to those of miR-564 overexpression, and miR-564 phosphorylates AKT and ERK1/2. This data suggest that miR-564 can serve as a prognostic marker for HCC and that it may be a therapeutic target for HCC.

Recently, an increasing number of studies have confirmed that abnormal miRNA expression is closely related to the occurrence and development of cancer [17, 18] and that a variety of miRNAs are involved in the development of liver cancer [19–22]. miR-564 was found to be downregulated in leukemia [23], lung cancer [14], glioma [16] and gastric cancer [13]. Yang *et al*. [14] analyzed 43 paired samples of lung cancer and adjacent noncancerous tissues and found that miR-564 was expressed at low levels in the lung cancer tissues; the authors also demonstrated that the expression of miR-564 was negatively correlated with TNM staging, and similar results were obtained by Cox proportional risk regression analyses. The results of Kaplan–Meier survival analyses showed that low levels of miR-564 expression in lung cancer predict poor prognosis. miR-564 expression in breast cancer [15] was also analyzed in different GEO databases using bioinformatics. According to the results, miR-564 was lowly expressed in high-grade, metastatic and progressive breast cancer, and low expression levels of miR-564 indicated a poor prognosis. To clarify the role of miR-564 in HCC, we collected 46 paired samples of HCC and adjacent tissues and measured miR-564 expression by qRT-PCR. Compared with adjacent tissues, miR-564 expression was significantly downregulated in HCC. Similar results were obtained by analyzing the GSE21362 database, and miR-564 expression levels were correlated with tumor size and metastasis. TCGA database analyses also revealed that low expression levels of miR-564 indicated a poor prognosis. This suggests that miR-564 may be involved in the development of HCC and that miR-564 may be a potential indicator of HCC prognosis.

Recent studies have indicated that miRNAs play a dual role, as they can both inhibit tumors and promote cancer. The expression levels of miR-107, miR-25 and miR-425-5p are elevated in HCC, and these miRNAs promote the progression of HCC [24–26]. The expression levels of miR-885-5p, miR-874-3p and miR-33a-3p are downregulated in HCC, and these miRNAs suppress HCC invasion and metastasis [27–29]. miR-564 has been confirmed to be significantly downregulated in glioma cell lines. Moreover, miR-564 overexpression inhibits glioma cell proliferation, invasion and metastasis *in vivo* and *in vitro*, and similar effects were observed in HCC. We constructed a lentiviral vector plasmid overexpressing miR-564 and showed that miR-564 overexpression significantly inhibited hepatocarcinoma cell proliferation. To further verify these results, we demonstrated that miR-564 overexpression inhibited subcutaneous tumor growth in nude mice. Invasion and metastasis are the most important manifestations of advanced liver cancer and are likely the main reasons why HCC is associated with a poor prognosis. In this study, we used Transwell assays to confirm that miR-564 overexpression significantly inhibits HCC cell invasion and metastasis.

miRNAs are important regulatory molecules in the body. miRNAs do not encode proteins, but they regulate the expression of target genes by complementarily binding with the 3′-UTR of mRNA, thereby regulating various physiological and pathological processes in the organism [30]. Several studies have demonstrated that miR-564 regulates the expression of target genes in various cancers. For example, miR-564 regulates the expression of TGF-β1 to suppress glioma cell proliferation and invasion [16], and miR-564 plays a role in the regulation of E2F3 in gastric cancer [13]. These results suggest that the regulatory mechanisms may be different in various tumors. In our study, we used bioinformatics software to predict the possible target genes of miR-564. The results suggested that the GRB2 3′-UTR contains a miR-564 binding site; consequently, GRB2 may be a target gene of miR-564. The results of the luciferase reporter assays showed that miR-564 specifically binds to the 3′-UTR of GRB2 mRNA. qRT-PCR and Western blot analysis showed that miR-564 overexpression inhibits GRB2 expression at the mRNA and protein levels; these results suggest that GRB2 is a direct target gene of miR-564.

**Figure 7:**
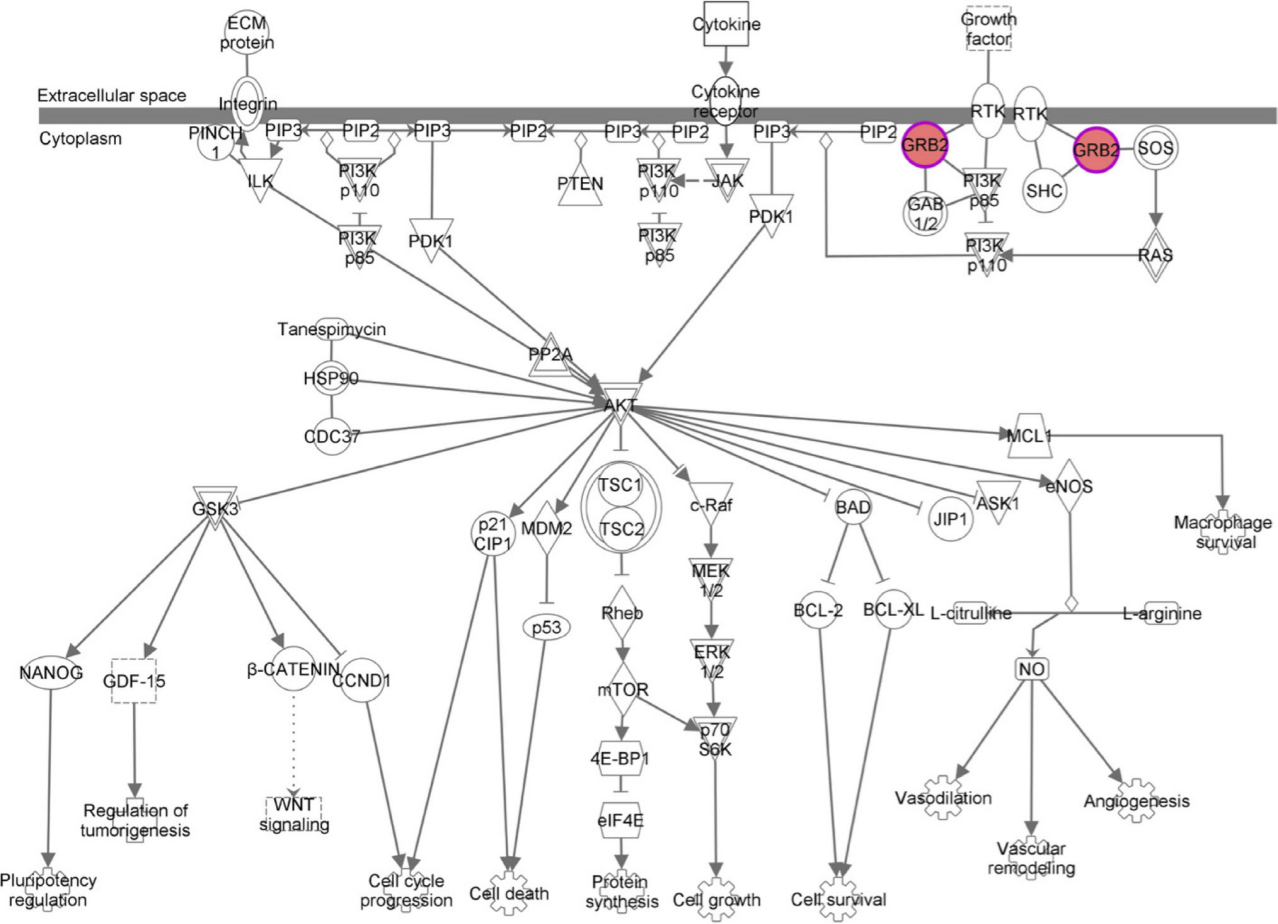
The GRB2 signaling pathway

GRB2 belongs to a class of adapter proteins that is widely expressed in cells; GRB2 is composed of an intermediate SH2 (Src homology 2) domain and two SH3 (Src homology 3) domains that are located at both ends [31, 32]. Recently, studies have shown that GRB2 is highly expressed in bladder cancer [33], breast cancer [34] and esophageal cancer [35] and that GRB2 is closely related to poor prognosis. GRB2 has also been shown to be highly expressed in HCC [36]; our study was consistent with this report. GRB2 was negatively associated with the expression level of miR-564, and GRB2 overexpression was closely related to the prognosis of HCC. Because of its structural characteristics, GRB2 has been found to promote the development of tumors via two pathways (Figure 7). First, GRB2 binds with SOS to form a complex. This complex is shuttled to the vicinity of the Ras protein near the cell membrane. SOS converts GDP to GTP through catalytic exchange; therefore, at the cell membrane SOS activates its substrate Raf kinase, which regulates MEK, MAPK and downstream protein kinase and transcription factors [37, 38]. Second, GRB2, in conjunction with its adaptor protein Gab1, is involved in the PI3K/AKT signaling pathway [39]. To further clarify the role and mechanism of the miR-564-GRB2 axis in HCC, we examined the expression of molecules downstream of GRB2 and found that miR-564 overexpression inhibited the phosphorylation of ERK1/2 and AKT and inhibited the expression of GRB2. When the expression of GRB2 was restored, ERK1/2 and AKT phosphorylation levels were increased. These results suggest that miR-564 overexpression may inhibit MAPK and PI3K/AKT pathway activation by modulating the expression of GRB2; this mechanism is similar to that in breast cancer. Mutlu *et al*. [15] found that miR-564 inhibited the activation of PI3K/AKT and MAPK by suppressing the expression of AKT2, GNA12, GYS1 and SRF. Many studies have indicated that ERK1/2 and AKT are involved in EMT in hepatocarcinoma cells [40, 41]. In this study, we found that miR-564 overexpression upregulated the protein levels of the epithelioid-like molecular marker E-cadherin and downregulated the levels of interstitial cell molecular markers N-cadherin and vimentin. These data suggest that miR-564 overexpression inhibits EMT in hepatocarcinoma cells by targeting the GRB2-ERK1/2-AKT axis.

In summary, we found that miR-564 inhibited HCC proliferation, invasion and metastasis by targeting the GRB2-ERK1/2-AKT axis, thereby inhibiting the malignant phenotype of liver cancer. However, our study has some limitations. First, GRB2 is likely only one of the many target genes of miR-564; therefore, there may be many regulatory channels that have not yet been explored. Second, our study included a relatively small cohort of patients and a short follow-up period. However, our study shows that miR-564 may be a marker for liver cancer prognosis and that it may be a new therapeutic target for HCC.

## MATERIALS AND METHODS

### Ethics statement

All patients in the study provided written informed consent. This study was approved by the Ethics Board of the Institute of Beijing Tongren Hospital and complied with the Declaration of Helsinki.

### Human specimens and cell culture

HCC tissues and corresponding adjacent noncancerous tissues were collected at the Department of General Surgery at Beijing Tongren Hospital of Capital Medical University. A total of 46 patients underwent radical resection for HCC from January 2015 to September 2016. HCC was confirmed by pathologic examination. All tissue specimens were immediately frozen in liquid nitrogen and then stored at −80°C. Prior to surgery, no patients received chemotherapy, radiotherapy, biological treatment or other treatment measures. The collected clinical data included sex, age, tumor size, alpha-fetoprotein (AFP) level, tumor number, HBsAg status, vein invasion status, tumor differentiation and tumor staging. The tumor stage was evaluated according to the seventh edition of the tumor, node, and metastasis (TNM) staging system (2010). This study was approved by the Beijing Tongren Hospital Ethics Committee. All specimens were obtained from the patients after informed consent had been signed. HepG2, Huh-7, and BEL-7402 cell lines were obtained from the Cell Bank of Shanghai Institute of Biochemistry & Cell Biology (Shanghai, China). MHCC97H and SMCC7721 cell lines were obtained from the Cell Bank of Zhongshan Hospital of Fudan University (Shanghai, China). Hep3B and LO-2 cell lines were obtained from the Cancer Hospital of the Chinese Academy of Medical Sciences (Beijing, China). Cells were maintained in Dulbecco's modified Eagle's medium (DMEM) supplemented with 10% fetal bovine serum (Gibco, Waltham, MA USA), 100 U/ml penicillin, and 100 μg/ml streptomycin (Invitrogen, Carlsbad, CA, USA) in 5% CO_2_ at 37°C.

### Plasmids and cell transfection

Lentiviral plasmids encoding miR-564 or a negative control were designed and manufactured by BioREE (Beijing, China). MHCC97H and SMCC-7721 cells were grown in 6-well plates to 20–30% confluence, and the culture medium was replaced with a transfection-enhancing solution containing lentivirus at a multiplicity of infection (MOI) of 30 and 50 μg/ml polybrene. After 12 h, the culture medium was replaced with complete medium for 72 h. Then, 1 μg/ml puromycin was administered for 2 weeks to select the cells, and the cells were harvested for subsequent studies.

For transient transfections, HCC cells were transfected with miR-564 mimic; miR-564 negative control (miR-NC); siRNA for GRB2, as shown in Table 1 for GRB2; GRB2 negative control siRNA (si-NC) (BioREE, Beijing, China); pcDNA3.1-GRB2 plasmid lacking the 3′-UTR; or pcDNA3.1-GRB2-NC (BioREE, Beijing, China) using Lipofectamine 2000 (Invitrogen) in accordance with the manufacturer's instructions. After 12 h, the culture medium was replaced with complete medium for 36 h before harvesting for other experiments.

**Table 1:** Sequences of human GRB2 shRNAs

No.	shRNAs targeting GRB2
siRNA-GRB2-1	5′-CCAGGCCCTCTTTGACTTT-3′
siRNA-GRB2-2	5′-CCAGAAACCAGCAGATATT-3′
siRNA-GRB2-3	5′-GCTTCATTCCCAAGAACTA-3′
siRNA-NC	5′-UUCUCCGAACGUGUCACGUTT-3′

### Quantitative real-time polymerase chain reaction (qRT-PCR)

Total RNA was extracted from cultured cells or tissues using TRIzol (Life Technologies, Carlsbad, CA, USA) qRT-PCR primer information was showed in Table 2. According to the manufacturer's instructions (Applied Biosystems, Foster City, CA, USA), miR-564 expression was measured by qRT-PCR using the TaqMan microRNA assay reverse transcription kit. U6 small nuclear RNA expression was used as an internal control. Real-time PCR was repeated in triplicate using a Real-Time PCR System (Applied Biosystems, Foster City, CA, USA). To determine the expression of various genes, the qRT-PCR reaction mixture was prepared according to the manufacturer's instructions (BIO-RAD, Beijing, China), and GAPDH expression was used as an internal control. The CT difference between the internal control and the target gene is presented as −ΔCT. ΔΔCT is the difference between the ΔCT values of paired specimens. 2ΔΔCT indicates the exponential value of ΔCT, and this value indicates the change in expression.

**Table 2:** qRT-PCR primer information

No.	Sequence
U6	Forward: 5′-CTCGCTTCGGGCAGCACA-3′
	Reverse: 5′-AACGCTTCACGAATTTGCGT-3′
GRB2	Forward: 5′-GGGCCTTTCTTATCCGAGA-3′
	Reverse: 5′-TGCACATCGTTTCCAAACTT-3′
GAPDH	Forward: 5′-AGCCACATCGCTCAGACAC-3′
	Reverse: 5′-GCCCAATACGACCAAATCC-3′

**Table 3:** Correlation between miR-564 expression and clinicopathological characteristics in 46 patients with HCC

Characteristics	*n*	miR-564 expression		*p*-values
		High	low	
All case	46	23	23	
Gender				0.32
Male	33	15 (45.5%)	18 (54.5%)	
Female	13	8 (61.5%)	5 (38.5%)	
Age				0.76
≥60	17	8 (47.1%)	9 (52.9%)	
<60	29	15 (51.7%)	14 (48.3%)	
HBsAg				0.32
Positive	33	15 (45.5%)	18 (54.5%)	
Negative	13	8 (61.5%)	5 (38.4%)	
Tumor size (cm)				0.001
≥5	21	5 (23.8%)	16 (76.2%)	
<5	25	18 (72.0%)	7 (28.0%)	
Tumor number				0.044
Single	34	20 (58.8%)	14 (41.2%)	
Multiple	12	3 (25.0%)	9 (75.0%)	
Differentiation				0.14
High	25	15 (60.0%)	10 (40.0%)	
Low	21	8 (38.1%)	13 (61.9%)	
Vein invasion				0.003
Yes	20	5 (25.0%)	15 (75.0%)	
No	26	18 (69.2%)	8 (30.8%)	
AFP(ng/ml)				0.47
>400	10	4 (40.0%)	6 (60.0%)	
≤400	36	19 (52.8%)	17 (47.2%)	
TNM stage				0.077
I–II	24	15 (62.5%)	9 (37.5%)	
III–IV	22	8 (36.4%)	14 (63.6%)	

### Cell proliferation, colony formation, transwell invasion and wound healing assays

Transfected MHCC97-H cells or SMCC-7721 cells were seeded onto 96-well plates, and cell proliferation was measured at 1, 2, 3, 4 days by the Cell Counting Kit 8 assay (Dojindo, Kumamoto, Japan) according to the manufacturer's instructions. For the colony formation assay, 300 transfected HCC cells were seeded onto a 6-well plate and cultured for 14 days in DMEM supplemented with 10% FBS. Then, the colonies were fixed in 95% ethanol and stained with a 4 g/L crystal violet solution. Colonies containing over 50 cells were counted. Cell invasion was detected using Transwell chambers (8-μm pore size; Millipore) with Matrigel (BD Biosciences, San Jose, CA, USA). In brief, 600 μl of complete medium was added to the bottom chamber. The transfected cells were suspended in serum-free medium, and 200 μl of the cell suspension (4 × 10^4^ cells) was placed in the upper chamber. After 24 h, the cells on the top surface of the membrane were removed using a cotton swab, and the cells on the bottom surface of the membrane were fixed in 95% ethanol and stained with a 4 g/L crystal violet solution. Cells adhering to the bottom surface of the membrane were counted in five randomly selected fields at 200x magnification. For the wound healing assay, cells were seeded onto six-well plates at 90% confluence and incubated overnight to allow adherence. A wound was then made along the center of each well by scratching the cell layer with the tip of a 200-μL pipette. Next, the wells were washed twice with phosphate-buffered saline (PBS) to remove any loose cells, and fresh medium was added. Photographs were taken at 0 and 24 h to assess cell migration into the wound. Each experiment was repeated three times.

### Xenograft model in nude mice

Animal studies were conducted in accordance with the University's Institutional Animal Care and Use Committee Guidelines. Six-week-old BALB/c nude mice were subcutaneously injected in the left flank with 6 × 10^6^ MHCC97H cells stably expressing miR-564 or miR-NC. The mice were also injected with an empty vector in the right flank. The mice were sacrificed according to a standard procedure after 28 days, and the tumors were harvested and weighed. The volume of each tumor was calculated according to the formula V = a × b × (a + b)/2, where a and b are the length and width of the tumor, respectively, measured by a sliding caliper.

### Western blot analysis

Equal amounts of protein were separated by 10% SDS-PAGE and then transferred to polyvinylidene fluoride (PVDF) membranes (Millipore, Bedford, MA, USA). Non-specific protein interactions were blocked by incubation with 3% non-fat milk in tris-buffered saline with Tween-20 (TBST) at 4°C for 1 h. Subsequently, the membranes were incubated for 2 h with antibodies against GRB2 (1:1,000, Abcam, Cambridge, UK), anti-p-AKT (Abcam, Cambridge, UK), anti-AKT (Abcam, Cambridge, UK), anti-ERK1/2 (Cell Signaling Technology CST, Danvers, MA, USA), anti-p-ERK1/2 (CST), anti-N-cadherin (CST), anti-E-cadherin (CST), anti-vimentin (CST) and anti-GAPDH (Abcam, Cambridge, UK). Next, the membranes were incubated with fluorescent secondary antibodies. GAPDH was used as the endogenous control.

### Luciferase reporter assay

293T cells were seeded onto a 24-well plate and co-transfected with luciferase reporter constructs encoding the wild-type 3′-UTR of GRB2 (GRB2-WT-3′-UTR) or a mutated 3′-UTR of GRB2 (GRB2-MUT- 3′-UTR) (RiboBio, Guangzhou, China) and miR-564 mimic or miR-564 mimic-NC using Lipofectamine 2000 (Invitrogen). After the cells were incubated for 48 h, they were washed with PBS and lysed with Passive Lysis Buffer (Promega). Firefly and *Renilla* luciferase activities were measured using a dual-luciferase reporter assay kit (Promega) according to the manufacturer's instructions. All experiments were repeated three times.

### miRNA and gene expression data

miRNA and gene expression data were collected from the GEO database (www.ncbi.nlm.nih.gov/gds, GSE21362 and GSE14520). After quality control, 73 HCC samples and adjacent non-tumor liver samples were used for miRNA analyses; 222 HCC samples and 212 normal liver samples were used for gene analyses.

### Identification of differentially expressed miRNAs and genes

The identification of differentially expressed miRNAs and genes in HCC was performed using the Limma package on the R platform and the downloaded miRNA and gene expression data mentioned above. The cutoff value that indicated significantly differentially expressed miRNAs and genes was a *P* value < 0.01 (Student's *t*-test) and an |lgFC| > 1 (fold change).

### Prediction of miRNA-target interactions

Experimentally validated miRNA-target interactions were collected from the miRTarBase database (release 6.0). Additional miRNA-target interactions were predicted using DIANA and TargetScan. miRNA-target interactions supported by evidence from at least one bench experiment or two prediction methods were selected for further analysis.

### Kaplan–Meier survival analysis

The survival of HCC patients was analyzed using data downloaded from The Cancer Genome Atlas (TCGA) database. A total of 130 and 180 HCC patients were included in the survival analyses of miR-564 and GRB2. For the miR-564 analysis, 130 patients were divided into two groups, the miR-564-low and miR-564-high groups, based on their miR-564 expression levels. Similarly, for the GRB2 analysis, 180 patients were divided into two groups, the GRB2-low and GRB2-high groups, based on their GRB2 expression levels. The miRNA and gene expression levels were measured using the Limma package on the R platform. The cutoff threshold was set to separate the top 10% ‘miR-564-low’patients from the 90% ‘miR-564-high’ patients, or 10% ‘GRB2-high’patients from the 90% ‘GRB2-low’ patients, respectively. The survival curves were estimated using the Kaplan–Meier method and compared with the log-rank test.

### Statistical analysis

Statistical analyses were performed using GraphPad Prism 6 (GraphPad Software, La Jolla, USA) and SPSS 16.0 (IBM, NY, USA) software. All experiments were conducted in triplicate, and the data are presented as the mean ± standard error of the mean (SEM). Paired-sample *t*-tests were used to compare the expression levels of miR-564 between tumor tissue and adjacent non-tumor tissues. The Kaplan–Meier method was used for overall survival analyses, and differences in survival curves were compared using the log-rank test. *P <* 0.05 was considered statistically significant.
